# Bronchoalveolar lavage is an ideal tool in evaluation of local immune response of pigs vaccinated with *Pasteurella multocida* bacterin vaccine

**DOI:** 10.14202/vetworld.2015.438-442

**Published:** 2015-04-07

**Authors:** Shiney George, Nagendra Nath Barman, Anjan Jyoti Nath, Bhupen Sarma

**Affiliations:** 1 North Eastern Regional Disease Diagnostic Laboratory, Government of Assam, Khanapara, Guwahati - 781 022, Assam, India; 2 Department of Microbiology, College of Veterinary Science, Khanapara, Guwahati - 781 022, Assam, India; 3 Pasteur Institute of India, Coonoor - 643 013, Nilgiris, Tamil Nadu, India; 4 Department of Surgery and Radiology, College of Veterinary Science, Assam Agricultural University, Khanapara, Guwahati - 781 022, Assam, India

**Keywords:** bronchoalveolar lavage, cell types, isotype specific enzyme linked immunosorbent assay, *P. multocida*, pig, pulmonary immune response

## Abstract

**Aim:**

The aim was to study the bronchoalveolar lavage (BAL) technique in evaluating the local immune response of pig immunized with *Pasteurella multocida* bacterin vaccine.

**Materials and Methods:**

Weaned piglets were immunized with formalin-inactivated P_52_ strain of *P. multocida* bacterin and evaluated for pulmonary immune response in BAL fluid. BAL was performed before vaccination and at different post vaccination days. The BAL fluid was assayed using enzyme-linked immunosorbent assay to study the development of *P. multocida* specific antibody isotypes and also evaluated for different cell populations using standard protocol.

**Results:**

The average recovery percentage of BAL fluid varies from 58.33 to 61.33 in vaccinated and control group of piglets. The BAL fluid of vaccinated pigs showed increase in antibody titer up to 60^th^ days post vaccination (8.98±0.33), IgG being the predominant isotype reached maximum titer of 6.12±0.20 on 45^th^ days post vaccination, followed by IgM and a meager concentration of IgA could be detected. An increased concentration of the lymphocyte population and induction of plasma cells was detected in the BAL fluid of vaccinated pigs.

**Conclusion:**

Though intranasal vaccination with *P. multocida* plain bacterin vaccine could not provoke a strong immune response, but is promising as lymphocyte population was increased and plasma cells were detected. BAL can be performed repeatedly up to 3/4 months of age in pigs to study pulmonary immune response without affecting their health.

## Introduction

Bronchoalveolar lavage (BAL) is a procedure in which a bronchoscope is passed through the mouth or nose into the lungs and fluid is squirted into a small part of the lung and then recollected for examination. BAL is a valuable tool for the diagnostic evaluation of animals with lower respiratory tract disease and also used for cytological, immunological, as well as microbiological analysis [1,2].

The respiratory system of many animal species including pigs is inhabited with commensal organism *Pasteurella multocida*, which is considered opportunistic pathogens associated with diseases such as atrophic rhinitis [3] and pneumonic pasteurellosis [4] in pigs. The systemic immune response against such infection has been studied extensively. However, it is important to understand simultaneously the role played by the local immune response in lower respiratory tract diseases, for the interplay of non-specific innate immunity and specific humoral and cellular immunity plays a major role in defense mechanism of the upper respiratory tract and pulmonary parenchyma [5].

To study the local immune response of the respiratory organs, BAL is a valuable tool. However, in animals, especially pigs, because of the typical anatomy of the larynx, collection of BAL becomes difficult and so earlier the animals had to be sacrificed at each schedule for collection of BAL [6].

The purpose of the present study was to design a method of repeated collection of BAL from live pigs without affecting their health and to evaluate the local immune response in BAL fluid of pigs vaccinated with *P. multocida* bacterin vaccine.

## Materials and Methods

### Ethical approval

The use of the animals for the present study was approved by Institutional Animal Ethics Committee and maintained as per the guidelines of the committee for the purpose of control and supervision of experiments on animals.

### Materials studied

The study materials consisted of BAL fluid samples of pigs before and after immunization with bacterin derived from reference P_52_ strain of *P. multocida*. BAL fluid was also collected from unvaccinated control group of pigs.

### Study area

This study was conducted in weaned piglets maintained in the Department of Microbiology, College of Veterinary Science, Khanapara, Assam.

### Study methods

Formalin inactivated bacterin of the reference P_52_ strain of *P. multocida* was prepared and its safety and sterility determined as per standard methods and the bacterial concentration was finally adjusted to 10^9^ organisms per ml of the suspension [7]. A total of 10 weaned piglets of same age group (2 months) and belonging to either sex were randomly divided into two groups consisting of 5 piglets in each group. Piglets in Group I were vaccinated with formalin-inactivated bacterin vaccine via the intranasal route @ 5 ml. Animals in Group II were kept as unvaccinated control and were instilled with 5 ml sterile normal saline, intranasally.

**Figure-1:**
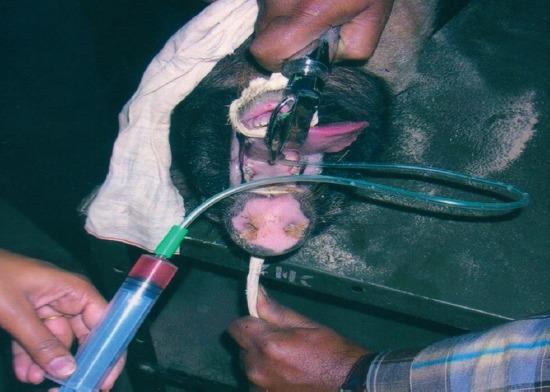
Bronchoalveolar lavage collection.

BAL fluid was collected from animals of both groups on day of vaccination and at 30, 45, 60, 90 and 120 days post-primary vaccination under anesthetic condition using a laryngoscope. Figure-1 shows method of performing BAL. Approximately 10 ml of sterile RPMI-1640 (Hi-Media Lab. Pvt. Ltd.) was infused into the lungs and was gently aspirated out after 2-3 minutes using a sterile syringe and a suction catheter (size 12 mm, Richdel). The lavage fluid was filtered through sterile gauze to remove mucus clumps and was centrifuged at 600 g for 15 min. The supernatant was collected in a sterile vial and stored at −20°C for determination of *Pasteurella* specific antibody by indirect enzyme-linked immunosorbent assay (ELISA). The sediment was washed thrice in sterile RPMI-1640 and re-suspended in little volume of RPMI-1640. This suspension was processed for counting total cells and differential leukocytic cell count.

Total leukocytic count was done at day of immunization, as well as at 45 days of post-immunization using Neubauer counter, and cell concentration was expressed as cells x 10^6^/ml of retrieved BAL fluid as described by Wilkie and Markham [8]. For Differential Leukocytic count, approximately 10 µl of BAL fluid was placed in Alcian blue coated slide [9] and was allowed to adhere for 20-30 min in a wet box at room temperature. The slides were then washed in phosphate-buffered saline and fixed in cold 100% ethanol for 10 min. The slides were then stained with 10% Giemsa stain for 20 min as described by Gehrke and Pabst [10]. Approximately, 100 cells were evaluated for differential cell counts and cells were classified as macrophages, lymphocytes, neutrophils, and plasma cells.

### Statistical analysis

Statistical analysis was done as per the method described by Snedecor and Cochran [11] to evaluate the immune response on different days post vaccination.

## Results

The method of collection of lavage used in live pigs was found to be free from any side effect. The mean ± standard error (SE) of recovered BAL fluids (in ml) was 5.8±1.66 and 6.1±0.66, with a recovery percentage of 58.33±1.66 and 61.33±0.66 in Group I and Group II piglets, respectively. On successive collection up to 120 days post vaccination, the yields were almost consistent to that of the first collection yield. All the animals remained clinically normal without showing any sign of the adverse reaction.

Pulmonary immune response was evaluated in BAL fluid at different days post vaccination by indirect ELISA and isotype-specific ELISA. The mean ± SE reciprocal ELISA titer in BAL fluids of both groups of pigs at different days post vaccination is shown in Table-1. On the day of vaccination, both the groups showed a detectable level of *Pasteurella* specific antibody in BAL fluid. In the vaccinated group (Group I), the whole antibody titer increased gradually and reached maximum value at 60^th^ days post vaccination, and marginally decreased thereafter. The unvaccinated control group (Group II), on the other hand, showed only detectable level of antibody titer throughout the study period. On statistical analysis, a significant difference (p<0.01) in ELISA titer in BAL fluid of vaccinated pigs at different days post-vaccination was observed. There was predominance of IgG immunoglobulin isotype in the BAL fluid of the pigs vaccinated with *Pasteurella* bacterin, which showed an increasing titer to reach maximum on 45^th^ days post vaccination, and decreased thereafter up to 120^th^ days post vaccination. IgM was detected at lower concentration following IgG, but the titer remained almost at the same level throughout the study period. Only a meager titer of IgA isotype could be detected, in both the groups.

**Table-1:** Mean±SE reciprocal whole antibody titer and isotype-specific ELISA titer (log 2 scale) in bronchoalveolar lavage of different groups of pigs at different days post vaccination.

Days post vaccination	Whole antibody titer		IgM		IgG		IgA	
			
	Group I	Group II	Group I	Group II	Group I	Group II	Group I	Group II
0	0.77^a^_A_±0.73	0.77^a^_A_±0.77	2.92^c^_B_±0.16	1.88^b^_A_±0.98	2.92^c^_B_±0.24	2.92^c^_B_±0.24	0.77^a^_A_±0.77	1.11^a^_A_±1.11
30	5.65^c^_B_±0.33	1.54^b^_A_±0.77	2.62^c^_B_±0.15	2.82^c^_B_±0.22	3.52^b^_C_±0.37	2.52^c^_B_±0.20	1.54^ab^_A_±0.77	1.55^ab^_A_±0.77
45	8.98^f^_E_±0.33	1.11^ab^_A_±1.11	2.62^c^_B_±0.15	2.72^c^_B_±0.22	6.12^e^_B_±0.20	2.72^c^_B_±0.24	1.88^b^_A_±0.98	1.11^a^_A_±1.11
60	8.65^f^_E_±0.33	0.77^a^_A_±0.77	2.65^c^_B_±0.33	2.62^c^_B_±0.15	5.92^e^_E_±0.24	2.72^c^_B_±0.24	1.55^ab^_A_±0.77	0.77^a^_A_±0.77
90	7.99^e^_D_±0.33	0.77^a^_A_±0.77	2.72^c^_B_±0.22	2.65^c^_B_±0.33	4.92^d^_D_±0.24	2.52^c^_B_±0.20	1.11^a^_A_±1.11	0.77^a^_A_±0.77
120	6.99^d^_C_±0.33	1.11^ab^_A_±1.11	2.65^c^_B_±0.33	2.62^c^_B_±0.15	3.92^cd^_C_±0.24	2.52^c^_B_±0.20	0.77^a^_A_±0.77	1.55^ab^_A_±0.77

Means in a row/column bearing a common superscript/subscript do not differ significantly

The mean ± SE of total leukocyte count (×10^6^) in BAL fluid of pigs (2.04±0.07, 2.37±0.15, 2.40±0.23, 2.40±0.12, 2.66±0.09, 2.44±0.15 and 2.16±0.14, 2.21±0.20, 2.30±0.10, 2.23±0.13, 2.12±0.18, 2.36±0.13 on 0, 30, 45, 60, 90, 120 days post vaccination for Group I and Group II respectively), however, did not show significant variation in vaccinated and unvaccinated pigs at different days post vaccination.

The pre-vaccination and post-vaccination mean ± SE differential leukocyte counts (%) in BAL fluid of pigs vaccinated with bacterins of *P. multocida* (Table-2), on the other hand, showed that the number of lymphocytes is comparatively higher in post-vaccinated BAL fluid than in the pre-vaccinated BAL fluid. Percentage of macrophages remained more or less constant before and after vaccination. No plasma cells were detected in pre-vaccinated BAL fluid, but after vaccination the percentage of plasma cells increased considerably. On the other hand, the neutrophil count in post-vaccination BAL fluid reduced as compared to pre-vaccination count. The rise in the level of lymphocytes and plasma cells corresponded to the proportionate decrease in the number of neutrophils in the post-vaccinated group.

**Table-2:** Pre and post-vaccination Mean±SE differential leucocyte counts (%) in bronchoalveolar lavage of pigs vaccinated with *Pasteurella multocida* bacterin.

Groups	Pre-vaccination counts (%)				Post-vaccination counts (%)			
	
	Lymphocyte	Macrophage	Plasma cell	Neutrophil	Lymphocyte	Macrophage	Plasma cell	Neutrophil
I	41.00±3.21	36.33±2.33	0.00±0.00	22.67±0.88	49.20±1.27	34.67±1.23	2.93±0.41	13.20±0.78
II	35.00±0.58	38.33±0.88	0.00±0.00	26.67±0.88	37.80±0.59	36.53±0.79	0.00±0.00	25.67±0.94

## Discussion

Since its introduction into clinical practice in the early 1980s, BAL has gained widespread acceptance as a clinical procedure that allows sampling of respiratory secretions with its leukocytes, other cellular components such as invading bacteria, acellular components such as cytokines, viral particles, and the microbial signatures such as proteins and nucleic acids [1]. Over years, it has been used as a tool of the diagnostic procedure in human and veterinary practices and research [1,2,12,13]. However, study carried out in most of the earlier experiments, animals had to be sacrificed at every/different schedule to collect BAL fluid. It might reflect variations in immune response. On the other hand, the method of collection of lavage used in live pigs, as described here, was found to be free from any side effect. The recovery of BAL fluid (%) was 58.33±1.66 and 61.33±0.66 in Group I and Group II, respectively. On successive collection up to 120 days post vaccination, the yields were almost consistent to that of the first collection yield. All the animals remained clinically normal without showing any sign of the adverse reaction. This method can be used successfully on several occasions to collect BAL from a single animal. Van Leengoed and Kamp [14] described a similar method of BAL in live pigs to isolate porcine alveolar macrophage and to quantitatively study the components of recovered lung fluid.

Though the method of BAL collection is free from adverse effects, some potential side effects includes cough, transient fever, transient chills and myalgias, transient infiltrates in most (resolves in 24 h), bronchospasm, transient fall of lung function, transient fall in baseline partial pressure oxygen in arterial blood etc. [15].

The pulmonary immune response studied in the BAL fluid with ELISA showed increase in the *Pasteurella* specific whole antibody titer and the isotype-specific ELISA indicated that IgG antibody predominates over IgM and there is meager quantity of IgA. Maintenance of a detectable level of antibody on the day of vaccination as well as in unvaccinated pigs clearly indicated that the animals were already primed against the commensal bacteria, which stimulated a low immune response. Majority of the antibodies present in the pulmonary parenchyma are locally produced and a small proportion are passively transferred across the hemato-alveolar barrier [16]. Further, the study confirmed that there was no fresh infection with *Pasteurella* throughout the period of study. Minute presence of secretary antibodies (IgA) in BAL showed that either local lymphoid tissues like Bronchi associated lymphoid tissue (BALT) was not developed or not activated by killed bacteria present in the vaccine. Study showed that the BALT is developed in response to chronic infection [17,18]. Therefore, the antigen in the form of bacterin instilled intranasally in lungs might have attracted the already primed IgG antibodies from the circulation towards the lungs showing a moderate rise in the IgG isotype in the BAL fluid. To activate the local humoral immune response in the pulmonary defense system, a new concept has therefore been suggested [17] to induce BALT and/or to increase its activity inducing M cells as a first step followed by inhaling killed bacteria as a vaccine in a second step.

The interplay of the non-specific innate immunity and specific humoral and cellular immunity plays a major role in defense mechanism of the respiratory system [5]. The BALT in the pig has been studied extensively to understand the role of the cellular defense mechanism, as well as the humoral response in terms of presence of immunoglobulin isotypes in BAL fluid. Macrophages, the major cellular constituent of non-specific pulmonary defense mechanism, are present normally in the alveolar lumen of healthy pigs with increased capability of phagocytosis [19], and are mostly consists of CD14+, CD163+, CD203α+, and MHCII+ phenotype [20]. Sarradell *et al*. [13] showed that pigs naturally infected with *Mycoplasma hyopneumoniae* showed a high morphologic and cellular organization of BALT. Macrophages and B lymphocytes were the main cellular components of germinal centers. T lymphocytes were primarily located in perafollicular areas of the BALT, lamina propria and within the airway epithelium, and plasma cells containing IgG or IgA at the periphery of the BALT, in the lamina propria of bronchi and bronchioles, in alveolar septa, and around bronchial submucosal glands. The hyperplastic BALT in porcine enzootic pneumonia cases consisted of macrophages, dendritic cells, T and B lymphocytes, and IgG and IgA plasma cells. CD4cells predominated over CD8 cells. Local humoral immunity appears to play an important role in the infection. In the swine infected with *P. multocida*, activated CD4+ and CD8+ T lymphocytes were found to concentrate in the perivascular and peribronchial regions of the lung [21].

## Conclusion

The intranasal vaccination of pigs with a *P. multocida* plain bacterin could not provoke a strong immune response, but seems promising as it could stimulate the lymphocytes and plasma cells in the pulmonary parenchyma as evaluated in the BAL fluid. To induce a robust local secretory immune response in the lower respiratory tract, probably actively multiplying attenuated vaccine candidate should be instilled prior to the vaccination with an inactivated *Pasteurella* bacterin vaccine. The BAL technique was found effective up to the 3/4 months age group of piglets under investigation. All the animals remained clinically normal and there was no other adverse reaction.

## Authors’ Contributions

SG: Planning and carrying out the study, sample processing and serological assay; NNB: Planning and carrying out the study, sample processing and serological assay; AJN: Sample processing and serological assay, data analysis, preparation and revision of the manuscript; BS: Planning and execution of BAL collection. All authors read and approved the final manuscript.
